# Exendin-4 Attenuates Hepatic Steatosis by Promoting the Autophagy-Lysosomal Pathway

**DOI:** 10.1155/2022/4246086

**Published:** 2022-07-15

**Authors:** Hsin-Hsien Yu, Hao-Chen Wang, Mao-Chih Hsieh, Ming-Che Lee, Bor-Chyuan Su, Yan-Shen Shan

**Affiliations:** ^1^Division of General Surgery, Department of Surgery, Wan Fang Hospital, Taipei Medical University, Taipei, Taiwan; ^2^Division of General Surgery, Department of Surgery, School of Medicine, College of Medicine, Taipei Medical University, Taipei, Taiwan; ^3^Institute of Clinical Medicine, College of Medicine, National Cheng Kung University, Tainan, Taiwan; ^4^Department of Anatomy and Cell Biology, School of Medicine, College of Medicine, Taipei Medical University, Taipei, Taiwan; ^5^Department of Surgery, College of Medicine, National Cheng Kung University, Tainan, Taiwan

## Abstract

Dysregulated hepatic steatosis may lead to chronic liver inflammation and nonalcoholic steatohepatitis (NASH). Recent studies have suggested that exendin-4, a glucagon-like peptide-1 agonist, may be a promising therapeutic for hepatic steatosis and NASH. However, the molecular mechanisms underlying the antihepatic steatosis actions of exendin-4 are not fully clear. Here, we demonstrate that autophagy is activated by either palmitic acid (PA) or oleic acid (OA) in HepG2 cells, and exendin-4 further enhances the autophagy-lysosomal pathway. We show that inhibition of autophagy by shLC3 attenuates exendin-4-mediated antisteatotic activity. Furthermore, expression of a key lysosomal marker, lysosome associated membrane protein 1 (LAMP1), is upregulated in OA + exendin-4-treated cells. The colocalization of LAMP1 and LC3 puncta further suggests that autophagic flux was enhanced by the cotreatment. Based on these findings, we conclude that autophagic flux might play an important role in the antisteatotic action of exendin-4.

## 1. Introduction

Nonalcoholic fatty liver disease (NAFLD) is the most common chronic liver disease in nearly all regions of the world [1]. Early-stage NAFLD typically begins as hepatic steatosis and can progress to more advanced forms, such as nonalcoholic steatohepatitis (NASH), with a prevalence of 5.7%–17% [2]. Furthermore, nearly 20% of patients with NAFLD develop liver cirrhosis [2]. Together, the liver-related complications of NAFLD result in a 10-year mortality rate of 30-40% [3]. In addition, more than 13% of hepatocellular carcinoma cases are related to NASH, and this condition is projected to become the leading indication for liver transplantation within a decade [4]. Current treatments for NAFLD include low-fat diet, low-calorie diet, Mediterranean diet, exercise, weight loss induction, vitamin E, and pioglitazone [5]. Clinical trials are underway to evaluate several other potential therapeutic agents, such as the peroxisome proliferator-activated receptor (PPAR) family PPAR*α*/*δ* ligand elafibranor [6], the bile acid analogue obeticholic acid [7], and the incretin pathway-modulating hormone glucagon-like peptide-1 (GLP-1) [8, 9]. However, the US Food and Drug Association has not yet approved any drugs specifically for the treatment of hepatic steatosis or NASH [10].

GLP-1 is released from L cells of the small intestine and acts on multiple tissues to reduce blood glucose levels [11]. For example, it targets pancreatic *β*-cells to stimulate insulin release and reduce glucagon production in response to various nutrients, neural signals, and endocrine factors [12]. In addition, GLP-1 and GLP-1 receptor agonists can modulate a broad range of physiological functions, including reduction of appetite and gastric emptying, cardioprotection, improvement of muscle or adipose tissue insulin sensitivity, and reduction of hepatic glucose production [13]. In patients with NAFLD, glucose stimulation is less effective at inducing GLP-1 secretion than it is in controls [14]. Moreover, reductions in hepatic GLP-1 receptor expression have also been observed in patients with NASH and in rats fed with a high-fat diet [15]. In light of this NAFLD-associated reduction in GLP-1 signaling, several potential mechanisms have been explored to understand how GLP-1 activity might ameliorate the condition. It is currently thought that the beneficial effects of GLP-1 may be associated with modulation of insulin signaling, oxidative stress, lipogenesis, and/or inflammatory cytokine expression pathways [16]. Moreover, the GLP-1 receptor agonist exendin-4 has been considered as a potential therapeutic agent for several metabolic disorders, such as type 2 diabetes [17], NASH [18], diabetic cardiomyopathy [19], and obesity [20]. While exendin-4 exhibits promising therapeutic potential for many metabolic disorders, it is still only approved for treatment of type 2 diabetes [17]. Notably, exendin-4 has been proposed to exert its antisteatotic effects via different pathways, such as reducing the expression of FABP-1 and FOXA1 [17] or inducing autophagy [21].

Autophagy is a catabolic self-digestion process that targets damaged organelles, unfolded proteins, or pathogens for degradation [22]. This self-renewal mechanism is thought to be critical for maintaining cellular energy and metabolic homeostasis in cells subjected to elevated levels of oxidative stress, nutrient deprivation, inflammatory cytokines, or even aging [22]. Recently, aberrant autophagy has also been reported to play vital roles in the pathogenesis of insulin resistance, obesity, and NAFLD [23]. Since autophagic flux is known to be impaired in NASH patients [24, 25], pharmacological or genetic modulation of autophagy may be a viable therapeutic strategy for patients with hepatic fat accumulation [22].

## 2. Materials and Methods

### 2.1. Steatotic Hepatocyte Model and Exendin-4 Treatment

The HepG2 human hepatocellular carcinoma cell line was used in this study. Cells were maintained in Dulbecco's modified Eagle's medium (Thermo Fisher Scientific) supplemented with 10% fetal bovine serum (Gibco), penicillin, and streptomycin (Thermo Fisher Scientific) at 37°C in a humidified atmosphere of 5% CO_2_. To establish a steatotic hepatocyte model, cells were treated with 250 *μ*M unsaturated fatty acids, oleic acid (OA; Sigma-Aldrich), or the saturated fatty acid palmitic acid (PA; Sigma-Aldrich) for 24 h. The four experimental groups comprised (1) vehicle treatment, (2) cells treated with exendin-4 (200 nM; Sigma-Aldrich), (3) cells treated with fatty acids OA or PA for 24 h, and (4) cells treated with OA or PA for 24 h followed by exendin-4 for another 24 h.

### 2.2. Immunofluorescence Staining

HepG2 cells were seeded on coverslips in 24-well plates and treated with OA or PA for 24 h followed by exendin-4 for another 24 h. Cells were fixed in cold methanol at -20°C for 10 min and washed with phosphate-buffered saline (PBS), then incubated with anti-LC3B and antilysosomal-associated membrane protein-1 (LAMP-1; Cell Signaling Technology) primary antibodies in Dako antibody diluent and placed in a cold room overnight. Cells were then incubated with fluorescent secondary antibodies and counterstained with DAPI (0.5 mg/mL, 1 : 2000 in PBS) for 1 min. Finally, cells were observed under fluorescence microscopy and photographed. Autophagic cells were defined as previously described with minor modifications [26]. Cells displaying more than 10 puncta were defined as autophagic cells.

### 2.3. Oil Red O Staining

To observe fat accumulation in the different hepatocyte treatment groups, Oil Red O (ORO) staining was performed. The staining solution was prepared by dissolving 0.5 g of ORO powder (Sigma-Aldrich) in 100 mL of 60% isopropyl alcohol and stored at 4°C away from light. Prior to staining, the stock solution was diluted with deionized water at a 3 : 2 ratio. Each coverslip was subjected to its respective treatment, then rinsed with PBS and stained with ORO. The intracellular lipid droplets were observed under light microscopy. Oil Red O staining was quantified as previously described [27].

### 2.4. LC3 Knockdown

HepG2 cells were transfected with short hairpin RNA (shRNA) against human MAP1LC3B (419388 and 428166; RNAi Core, Taiwan) for 48 h. The knockdown efficiency was determined by immunoblotting with anti-LC3B or antibody (Cell Signaling Technology). *β*-Actin served as an internal control. The *β*-actin antibody was purchased from Cell Signaling Technology. After successful knockdown of LC3, cells were incubated with OA or PA in the presence or absence of exendin-4. ORO staining was then performed to assess whether autophagic activation mediates the effects of exendin-4 on intracellular fat.

### 2.5. Statistical Analysis

Results are expressed as mean ± standard error of the mean. One-way or two-way ANOVA was performed with Tukey's post hoc multiple comparisons of individual groups. Significance was set at *P* < 0.05. Statistical analyses were performed using SPSS (version 18.0; IBM, USA).

## 3. Results

### 3.1. Exendin-4 Induces Autophagic Activity

**Figure 1:**
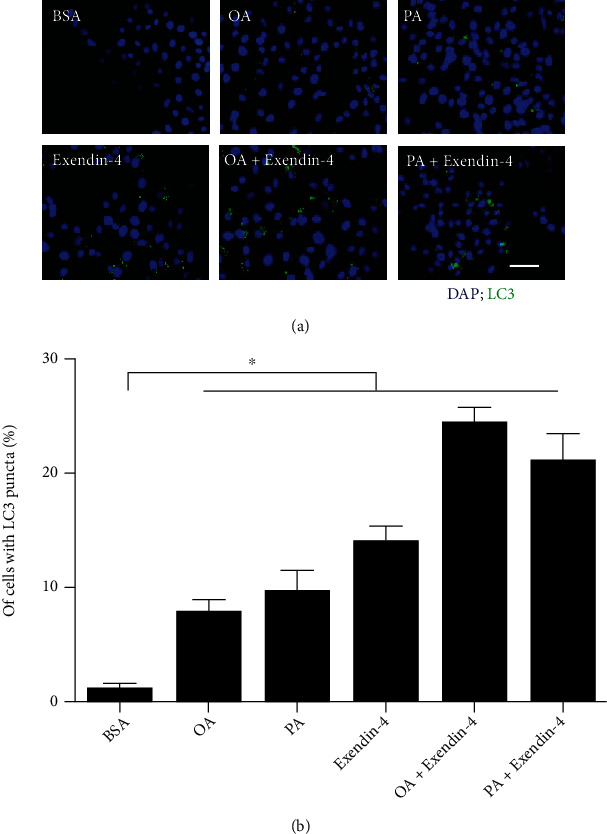
Exendin-4 induces autophagy. (a) HepG2 cells were treated with oleic acid (OA; 250 *μ*M) or palmitic acid (PA; 250 *μ*M) for 24 h. Then, cells were treated with or without exendin-4 (200 nM) for another 24 h. After treatment, cells were stained for LC3 using immunofluorescence. LC3 puncta formation was observed under fluorescence microscopy. Scale bar: 50 *μ*m. (b) Quantification of autophagic cells. ^∗^*p* < 0.05. All experiments were repeated three times with similar results.

**Figure 2:**
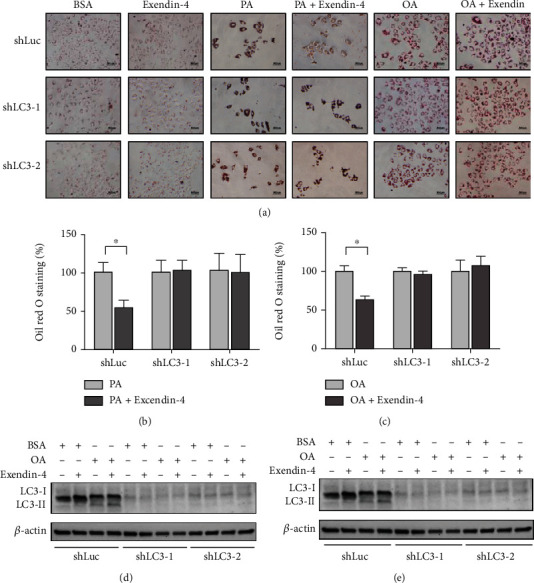
Exendin-4 attenuates unsaturated fatty acid-induced steatosis. (a) HepG2 cells were transfected with shLuc (nontargeting shRNA) or shLC3-1/shLC3-2 for 48 h. Subsequently, the cells were treated with OA (250 *μ*M) or PA (250 *μ*M) for 24 h. The cells were then treated with or without exendin-4 (200 nM) for another 24 h. Oil Red O staining was performed. (a–c) Quantification of Oil Red O signals. (b, c) Cells were treated as described in (a). Cell lysates were collected and immunoblotted with the indicated antibodies. All experiments were repeated three times with similar results.

To confirm that exendin-4 can induce autophagy in our *in vitro* system, HepG2 cells were treated with exendin-4. We found that the exendin-4 treatment markedly increased the number of LC3-positive puncta (Figures 1(a) and 1(b)) and LC3-II levels (Figures 2(d) and 2(e)), indicating elevated autophagy. In addition, both OA and PA treatments increased the numbers of LC3-positive puncta (Figures 1(a) and 1(b)) and LCE-II levels (Figures 2(d) and 2(e)). Interestingly, OA/PA-induced LC3-puncta accumulation (Figures 1(a) and 1(b)) and LC3-II levels (Figures 2(d) and 2(e)) could be potentiated by subsequent exendin-4 treatment.

### 3.2. Exendin-4 Alleviates OA/PA-Induced Lipid Droplet Accumulation via Upregulated Autophagy

Both OA and PA significantly increased numbers of lipid droplets in HepG2 cells, indicating that OA and PA treatments induce steatosis (Figures 2(a)–2(c)). Moreover, exendin-4 posttreatment alleviated the PA- and OA-induced lipid droplet accumulation, suggesting its antisteatosis action. Importantly, the antisteatosis effects of exendin-4 were abrogated when LC3 was silenced prior to the treatments (Figures 2(a)–2(c)). Of note, LC3 silencing efficiency was validated by Western blotting (Figures 2(d) and 2(e)). Together, these results suggest that exendin-4 suppresses OA/PA-induced steatosis via the induction of autophagy.

### 3.3. Exendin-4 Facilitates Autophagic Flux

**Figure 3:**
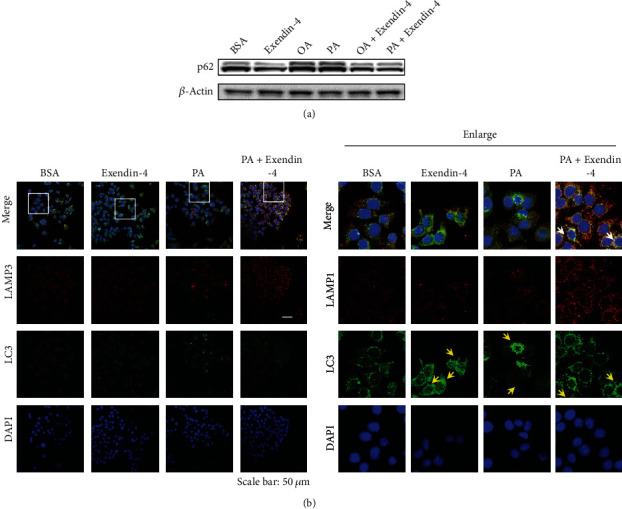
Exendin-4 enhances autophagic flux. (a) HepG2 cells were treated with oleic acid (OA; 250 *μ*M) or palmitic acid (PA; 250 *μ*M) for 24 h. Then, cells were treated with or without exendin-4 (200 nM) for another 24 h. Cell lysates were collected and immunoblotted with the indicated antibodies. (b) Cells were treated as described in (a). After treatment, cells were stained for LC3 and LAMP1 by immunofluorescence. Scale bar: 50 *μ*m. Yellow arrows indicate LC3 autophagic vacuoles. White arrows represent colocalized lysosome (LAMP1) with LC3 puncta. All experiments were repeated three times with similar results.

To verify the impacts of exendin-4 and PA on autophagic flux, cells were treated with exendin-4, PA, or the combination (PA + exendin − 4). In order to monitor autophagic flux, the protein levels of p62 were assessed by western blotting (Figure 3(a)). We found that p62 was not affected by OA and PA treatments. However, exendin-4 treatment reduced the level of p62. Furthermore, cells were treated as described and then stained for lysosomal-associated membrane protein 1 (LAMP-1; lysosomal marker) and LC3. In line with our other experiments, PA and exendin-4 treatments both increased the numbers of LC3-positive puncta (Figure 3(b)). In addition, PA + exendin − 4 exposure dramatically increased the level of LAMP1 staining. Strikingly, the LAMP1 signals were highly colocalized with LC3 puncta only in the PA + exendin − 4-treated cells (Figure 3(b)). These findings suggest that in addition to increasing the cellular autophagosome content, exendin-4 is able to enhance autophagic flux.

## 4. Discussion

In the present study, we investigated the role of autophagy in exendin-4-mediated antisteatotic activity. Using HepG2 cells, we showed that autophagy is activated by treatment with either fatty acids (OA and PA) or exendin-4 alone (Figure 1). In addition, the appearance of fatty acid-induced autophagic vacuoles is further enhanced by exendin-4 posttreatment (Figure 1). We also showed that LC3 knockdown abolishes exendin-4-mediated antisteatotic activity (Figure 2), suggesting that the induction of autophagy is required for the antisteatotic activity of exendin-4. Furthermore, exendin-4 enhances LAMP1 expression in PA-treated cells, suggesting that lysosomal activity is stimulated by the treatment (Figure 3). Colocalization of autophagosomes and lysosomes can be taken to mean that autophagosome-lysosome fusion has been initiated [28]. Since LAMP1 signals were colocalized with LC3 puncta and the level of p62 was reduced, we conclude that autophagic flux was induced by exendin-4. *In vivo* studies have shown that high-fat diet impairs autophagic flux and results in hepatic lipid accumulation [22]. Similar findings have also been reported in studies of NAFLD patients [29]. Notably, high fat diet-induced endoplasmic reticulum stress is also known to be more severe when autophagic flux is blocked, and this occurrence can accelerate the progression of NAFLD [22]. Here, we demonstrated that exendin-4 potentiates autophagic flux in fatty acid-loaded cells (Figure 3), and this action likely alleviates lipid-droplet accumulation (Figure 2). These findings are in line with a recent study that showed liraglutide, another GLP-1 receptor agonist, suppresses steatosis by inducing autophagic flux [30].

Exendin-4 has been shown to induce autophagy in many type of cells, including cardiomyocytes [31], pancreatic *β*-cells [32], and ovarian cancer cells [33]. In this study, we found that exendin-4 also induces autophagy in HepG2 cells (Figures 1 and 2); however, the underlying mechanisms remain to be determined. Based on other reports, exendin-4 might induce autophagy via mTOR-dependent or mTOR-independent pathways [31, 32].

Hepatic autophagy can provide amino acids, glucose, and free fatty acids for energy production and organelle renewal in starved cells [22, 24]. A growing body of evidence suggests that hepatic autophagy may also heavily influence other metabolic pathways, including glycogenolysis, gluconeogenesis, *β*-oxidation, and some specific homeostatic pathways [34]. Although autophagy was initially considered to be a nonselective bulk degradative process, more recent studies have reported that hepatic autophagosomes may selectively degrade specific cytosolic materials to regulate metabolic functions [34]. This type of autophagy (called selective autophagy) involves a process of cargo labeling, assembly of adaptor proteins, and transfer of targets to LC3/GABARAP proteins on the phagophore to form autophagosomes [35]. Singh et al. first demonstrated an interrelationship between autophagy and lipid metabolism in mouse liver; this process was initially called macrolipophagy and has been referred to as lipophagy in later literature [36]. In this type of selective autophagy, an autophagosome forms around part of a lipid droplet and fuses with a lysosome to stimulate degradation of the lipid droplet [36]. The physiological roles of lipophagy in the liver are thought to include the production of energy from fatty acid oxidation and also defense against harmful hepatic fat accumulation [37]. Based on our findings, we speculate that exendin-4 might activate lipophagy as a specific defense mechanism. Regardless of whether the autophagy pathway is selective or nonselective, our findings strongly suggest that exendin-4 exerts it antisteatotic effects by enhancing autophagic flux.

## Data Availability

The raw data supporting the conclusion of this article will be made available by the authors, without undue reservation.
