# Corrigendum to “Exendin‐4 Attenuates Hepatic Steatosis by Promoting the Autophagy‐Lysosomal Pathway”

**DOI:** 10.1155/bmri/9793841

**Published:** 2025-08-26

H.‐H. Yu, H.‐C. Wang, M.‐C. Hsieh, M.‐C. Lee, B.‐C. Su, and Y.‐S. Shan, “Exendin‐4 Attenuates Hepatic Steatosis by Promoting the Autophagy‐Lysosomal Pathway,” *BioMed Research International* 2022, (2022): 4246086, https://doi.org/10.1155/2022/4246086.

In the article, there is an error in Figure 2e, which was mistakenly duplicated from Figure 2d in the production process. Additionally, the Figure 2 legend contains errors. The correct Figure 2 and its accompanying legend is shown below:

Figure 2Exendin‐4 attenuates unsaturated fatty acid–induced steatosis. (a) HepG2 cells were transfected with shLuc (nontargeting shRNA) or shLC3‐1/shLC3‐2 for 48 h. Subsequently, the cells were treated with OA (250 *μ*M) or PA (250 *μ*M) for 24 h. The cells were then treated with or without Exendin‐4 (200 nM) for another 24 h. Oil Red O staining was performed. (b, c) Quantification of Oil Red O signals. (d, e) Cells were treated as described in (a); cell lysates were collected and immunoblotted with the indicated antibodies. All experiments were repeated three times with similar results.

**Figure:**
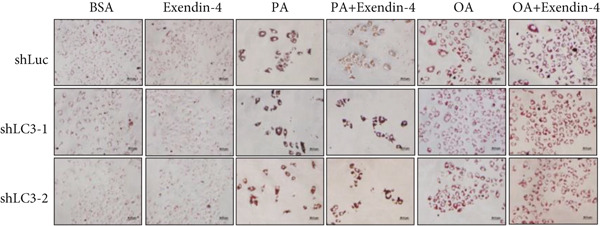
(a)

**Figure:**
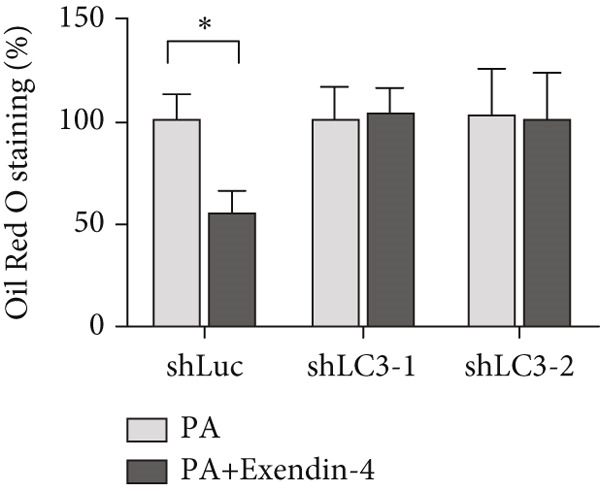
(b)

**Figure:**
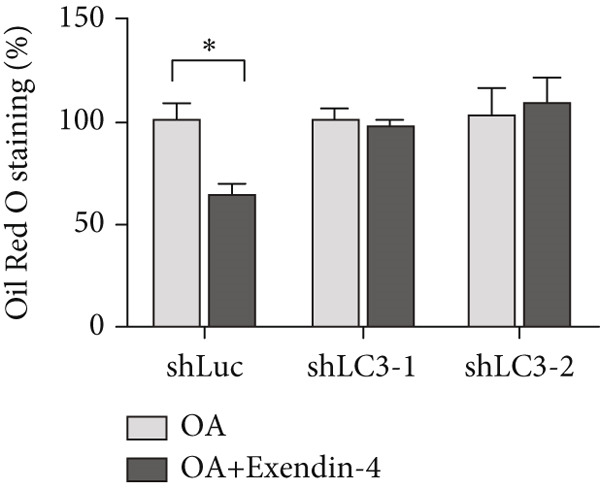
(c)

**Figure:**
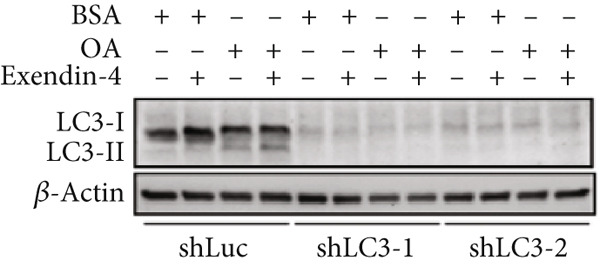
(d)

**Figure:**
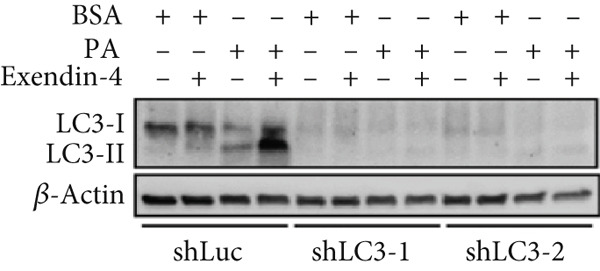
(e)

We apologize for this error.

